# MET Exon 14 Skipping Mutations in Lung Cancer: Clinical–Pathological Characteristics and Immune Microenvironment

**DOI:** 10.3390/curroncol32070403

**Published:** 2025-07-14

**Authors:** Qianqian Xue, Yue Wang, Qiang Zheng, Ziling Huang, Yicong Lin, Yan Jin, Yuan Li

**Affiliations:** 1 Department of Pathology, Fudan University Shanghai Cancer Center, Shanghai 200032, China; 19211230022@fudan.edu.cn (Q.X.); 15738819772@163.com (Y.W.); zq19931125@163.com (Q.Z.); zih2110@163.com (Z.H.); 21211230010@m.fudan.edu.cn (Y.L.); jinyan777_2014@163.com (Y.J.); 2 Department of Oncology, Shanghai Medical College, Fudan University, Shanghai 200032, China; 3 Institute of Pathology, Fudan University, Shanghai 200032, China

**Keywords:** MET exon 14 skipping mutation, non-small-cell lung cancer, immune checkpoints, immune microenvironment, multiplex immunofluorescence

## Abstract

MET exon 14 skipping mutations have emerged as significant driver alterations in non-small-cell lung cancer (NSCLC), contributing to tumor progression. This study examines the immune microenvironment in NSCLC patients with these mutations and its prognostic implications. We performed multiplex immunofluorescence (mIF) staining on formalin-fixed paraffin-embedded (FFPE) tissue samples from nine NSCLC patients, including four recurrent/metastatic and five non-recurrent/non-metastatic patients. Two panels assessed immune cell markers (CD8, CD4, CD20, CD68, and FoxP3) and immune checkpoints (PD-L1, LAG3, and TIM3). Immune cell infiltration and checkpoint expression were analyzed using HALO^TM^ software (version 3.6.4134.464). Nearest neighbor analysis was conducted to assess the proximity of immune cells to tumor cells. Univariate Cox regression analysis assessed factors associated with disease-free survival (DFS). CD8+TIM3+ and CD8+LAG3+ cells were predominantly located in the tumor parenchyma of recurrent/metastatic patients but localized to the stroma in non-recurrent/non-metastatic patients. Non-recurrent/non-metastatic patients exhibited a higher density of tertiary lymphoid structures and closer proximity of CD20+ B cells, CD8+TIM3+, and CD8+LAG3+ cells to tumor cells compared to recurrent/metastatic patients, though the differences were not statistically significant. Cox regression analysis suggested a potential association between higher densities of CD8+TIM3+ cells and improved DFS (HR = 0.89), though these findings did not reach statistical significance. Our findings suggest that differences in immune microenvironmental factors, particularly those related to immune checkpoint expression (TIM3 and LAG3), may influence clinical outcomes in NSCLC patients with MET exon 14 skipping mutations. Further studies are needed to validate these observations and explore potential therapeutic implications.

## Simple Summary

Lung cancer is a leading cause of cancer-related deaths, but new treatments targeting specific gene changes have improved outcomes for many patients. One such change, called MET exon 14 skipping, occurs in a small group of patients and is linked to more aggressive tumors. While targeted therapies have shown promise, the benefit of immunotherapy in these patients remains unclear. In this study, we used a special technique to examine the immune environment of tumors with this mutation. We looked closely at immune cells and how they interact with cancer cells. Our goal was to better understand why some patients respond to treatment while others do not. Although the number of cases in our study was limited, our findings offer early clues that may help guide future research, improve treatment strategies, and eventually lead to more personalized care for patients with this type of lung cancer.

## 1. Introduction

For patients harboring driver gene alterations in lung cancer, targeted therapies have significantly improved prognosis. Currently, an increasing number of driver genes are being identified. Among these, the MET exon 14 skipping mutation has garnered considerable attention as a newly recognized driver mutation. The MET gene encodes a receptor tyrosine kinase known as hepatocyte growth factor receptor (HGFR). This mutation results in the skipping of the 14th exon of MET mRNA, preventing MET protein degradation and increasing its stability. Consequently, this sustains activation of the MET signaling pathway, promoting tumor cell growth and dissemination [1,2,3].

The overall median frequency of MET exon 14 skipping mutations in non-small cell lung cancer (NSCLC) is approximately 2.0%, with slight variation across histological subtypes and minimal geographic differences [4]. These mutations are more frequently observed in elderly and non-smoking individuals [5] and are most commonly associated with the adenocarcinoma subtype in older patients [4]. While this mutation predominantly occurs in lung adenocarcinoma, it can also manifest in other types of NSCLC [3,4,6]. Due to its association with tumor invasiveness and metastasis, patients typically experience poorer outcomes [7].

Compared with conventional chemotherapy or immunotherapy, targeted therapies—specifically MET tyrosine kinase inhibitors (TKIs)—have shown superior clinical efficacy in patients harboring this mutation [4]. Agents such as capmatinib [8] and tepotinib [9] have demonstrated promising efficacy against this mutation, offering new therapeutic avenues for patients [10]. Recent clinical studies have expanded the therapeutic landscape for MET exon 14 skipping mutation NSCLC. Ensartinib has demonstrated notable clinical activity and acceptable tolerability in patients who have received prior treatments [11]. Likewise, findings from the phase 1 CHRYSALIS study indicate that amivantamab, a bispecific EGFR-MET antibody, provides durable antitumor activity in patients with MET exon 14 skipping advanced NSCLC, including those with prior resistance to MET therapies, while maintaining a manageable safety profile [12]. Additionally, lung cancers with MET exon 14 skipping mutations exhibit distinct characteristics in the immune microenvironment. Studies have indicated heightened levels of programmed death-ligand 1 (PD-L1) expression in these tumors [13,14], suggesting potential responsiveness to immune checkpoint inhibitors (ICIs) [15]. However, the clinical benefit of ICIs in this setting remains controversial. Although a substantial proportion of MET exon 14 skipping mutation NSCLC tumors express PD-L1, responses to PD-1 blockade have been infrequent, and the overall efficacy of immunotherapy in this population has been modest [14]. A recent study investigating first-line immunotherapy in MET exon 14 skipping mutation NSCLC demonstrated that combination chemo-immunotherapy was associated with prolonged progression-free survival (PFS), an increased objective response rate (ORR), and numerically improved overall survival (OS) compared with chemotherapy alone [16]. Notably, higher PD-L1 tumor proportion scores (TPSs) were not associated with better immunotherapy outcomes [16].

However, despite these advances, the immune landscape of MET exon 14-altered NSCLC remains incompletely understood. Given the limited response rates to immunotherapy observed in clinical practice, there is an unmet need to better characterize the tumor immune microenvironment in this subgroup. A deeper understanding may help identify predictive biomarkers and inform more effective treatment strategies for these patients.

In conclusion, this study will utilize multiplex immunohistochemical staining (mIHC) to provide a detailed characterization of the immune microenvironment in lung cancers harboring MET exon 14 skipping mutations. The mIHC technique allows for the simultaneous labeling and analysis of various immune cell populations and their associated molecular markers, offering a comprehensive understanding of immune cell distribution, subtypes, and interactions. This approach is anticipated to yield critical insights that could inform the development of novel therapeutic strategies. By thoroughly examining the immune microenvironment in these lung cancers, we intend to explore potential patterns that might be relevant to biomarker discovery and future therapeutic strategies. Although our current analysis is based on a limited number of cases, our findings provide preliminary insights and lay the groundwork for future studies. With expanded cohorts and more comprehensive data, it may become possible to identify clinically actionable immune signatures and develop personalized treatment approaches tailored to individual patients.

## 2. Methods

### 2.1. Patients

This study included 57 patients with primary NSCLC harboring MET exon 14 skipping mutations, who underwent surgical resection at Fudan University Shanghai Cancer Center between 2020 and 2022. All patients were treatment-naive prior to surgery. Among the 57 patients, nine received adjuvant chemotherapy with pemetrexed plus carboplatin or cisplatin following surgery. MET exon 14 skipping mutations were confirmed using either amplification refractory mutation system–polymerase chain reaction (ARMS-PCR) or next-generation sequencing (NGS). We retrospectively collected clinicopathological data, including gender, age, smoking history, tumor histology, TNM stage, intravascular carcinoma embolus, pleural infiltration, co-occurring mutations, PD-L1 TPS, and postoperative survival time. Tumor histology was independently verified by two expert pathologists. Clinical staging was based on the 8th edition of the American Joint Committee on Cancer (AJCC) staging system. Follow-up was conducted from February 2020 to May 2024. All patients provided written informed consent upon admission. This study was approved by the Ethics Committee of Fudan University Shanghai Cancer Center.

### 2.2. Multiplex Immunofluorescence (mIF)

We randomly selected 4 recurrent/metastatic patients and 5 non-recurrent/non-metastatic patients from the aforementioned study cohort. Among the recurrent/metastatic and non-recurrent/non-metastatic groups, one patient in each group received four cycles of adjuvant pemetrexed plus cisplatin following surgical resection. Formalin-fixed paraffin-embedded (FFPE) specimens from these 9 patients were sectioned into 4 μm thick paraffin slices and mounted on anti-detachment slides. The tissue sections were provided by the Department of Pathology, Fudan University Shanghai Cancer Center. Each sample underwent hematoxylin and eosin (H&E) staining as well as mIF staining using two distinct panels. The first panel included six markers, CD8, CD4, CD20, CD68, FoxP3, and Pan-CK, while the second panel comprised CD8, CD20, PD-L1, LAG3, TIM3, and Pan-CK. In situ multiplex staining was performed using tyramide signal amplification (TSA) in sequential rounds of staining.

The staining process was conducted as follows: First, the slides were baked at 60–65 °C for 2 h, followed by deparaffinization in xylene and rehydration through a graded ethanol series. Antigen retrieval was carried out using microwave heating in appropriate buffers. Ethylenediaminetetraacetic acid (EDTA) buffer (pH = 9.0) was used for CD4, CD20, FoxP3, PD-L1, LAG3, and TIM3, while sodium citrate buffer (pH = 6.0) was used for CD8, CD68, and Pan-CK. To block endogenous peroxidase activity, the sections were treated with 3% hydrogen peroxide for 10–15 min.

Each slide underwent multiple rounds of staining, involving incubation with specific primary antibodies, followed by secondary antibodies and the covalent binding of TSA fluorophores. After each round, antibodies were stripped using microwave treatment, allowing for subsequent rounds of staining. Upon completion of all staining cycles, the sections were counterstained with DAPI to visualize nuclei and mounted with an anti-fade medium. The slides were scanned, and all fluorescent signals were captured using the KR-HT5^TM^ high-throughput fluorescence digital pathology scanner (KR Pharmtech, Shanghai, China). Detailed information regarding the two staining panels can be found in Supplementary Tables S1 and S2.

### 2.3. Data Analysis

Statistical analysis and data visualization were performed using R software (version 4.3.3) and GraphPad Prism (version 10.2.0). Univariate and multivariate Cox regression analyses were performed using R version 4.4.0 (24 April 2024), along with Zstats 1.0 (www.zstats.net, accessed on 14 June 2024). All statistical tests were two-sided, with a *p*-value of <0.05 considered statistically significant.

## 3. Results

### 3.1. Clinicopathologic Characteristics

In Supplementary Table S3, we include 57 patients with *MET* exon 14 skipping mutations, confirmed by ARMS-PCR or NGS between 2020 and 2022. The majority of patients (71.9%) were over 60 years old, and the gender distribution was balanced (52.6% or *n* = 30 male). A significant proportion of the patients (64.9%) were never-smokers. The most common histological subtype was invasive adenocarcinoma (*n* = 45, 78.9%), followed by minimally invasive adenocarcinoma (*n* = 8, 14.0%), adenocarcinoma with sarcomatoid carcinoma (*n* = 2, 3.5%), adenocarcinoma in situ (*n* = 1, 1.8%), and squamous cell carcinoma (*n* = 1, 1.8%). The PD-L1 TPS was ≥1% in 23 patients (40.4%) and ≥50% in 14 patients (24.6%). T stage was predominantly T1 (82.5%), and N stage was predominantly N0 (86.0%). Pleural infiltration and intravascular carcinoma embolus were not common.

During the follow-up period, six patients experienced recurrence or metastasis. As shown in Table 1, compared to those without recurrence/metastasis, all patients with recurrence/metastasis were male, and the majority (83.3%) were former smokers (*p* < 0.05). The predominant T stage in this group was T2a (50%), and a significant portion (50%) had intravascular carcinoma embolus (*p* < 0.05).

**Figure 1:**
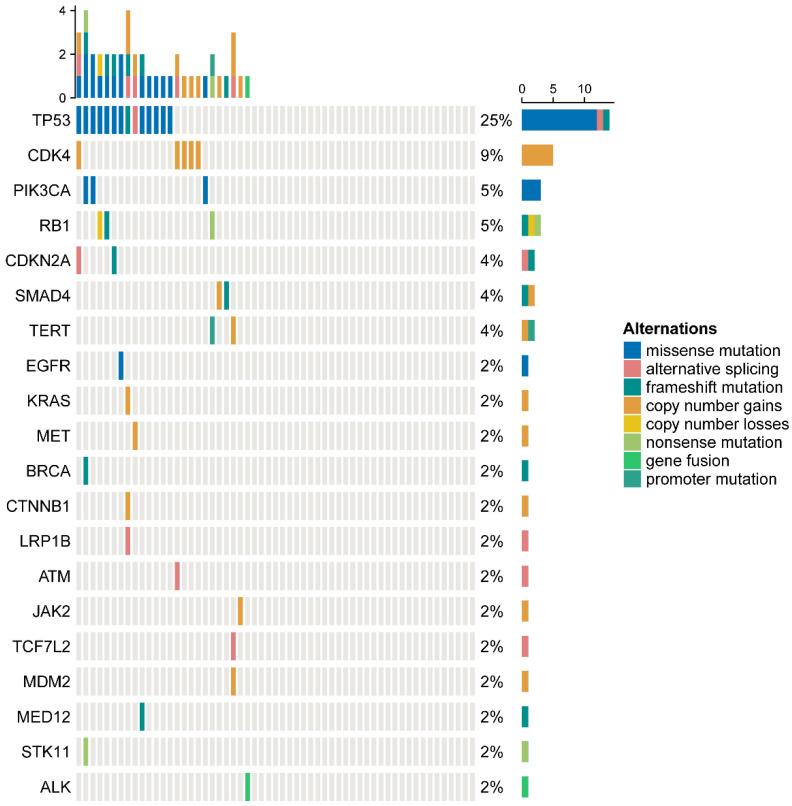
Co-mutation frequencies among patients with *MET* exon 14 skipping mutations.

Among the 57 patients, the most common co-mutation was TP53 (25%), followed by CDK4 (9%), PIK3CA (5%), RB1 (5%) and others, as detailed in Figure 1.

### 3.2. Infiltrating Immune Cells

**Figure 2:**
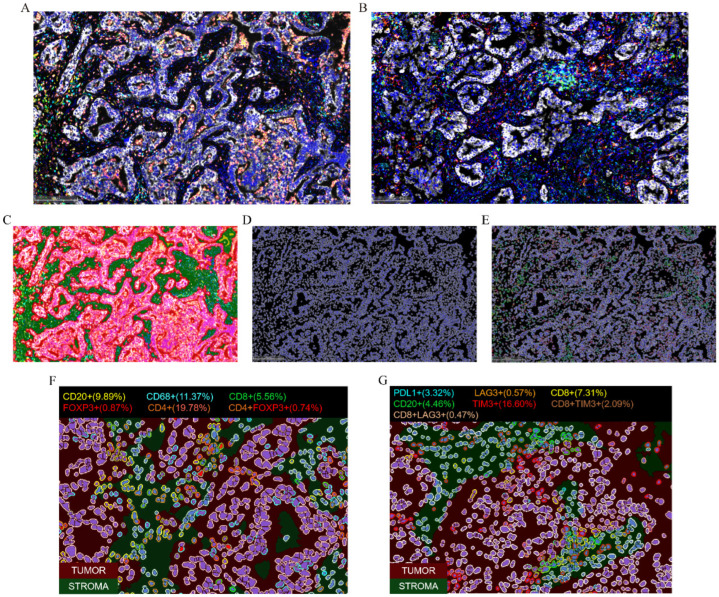
Multiplex immunofluorescence (mIF) staining results for a representative sample of Panel 1 (**A**) and Panel 2 (**B**). (**C**–**E**) Schematic of the analysis workflow. Region segmentation—red indicates tumor parenchyma, green indicates stroma (**C**); nuclear identification (**D**); and identification of positive cells in each channel (**E**). (**F**,**G**) Identification and quantification of various infiltrating immune cells in different regions for Panel 1 (**F**) and Panel 2 (**G**).

We randomly selected four recurrent/metastatic and five non-recurrent/non-metastatic patients for mIF staining to explore differences in the immune microenvironment between the two groups. Two staining panels were designed for this study. Panel 1 included the markers CD8, CD4, CD20, CD68, FoxP3, and Pan-CK, while Panel 2 included CD8, CD20, PD-L1, LAG3, TIM3, and Pan-CK. Figure 2A,B present the mIF staining results for one representative case.

The staining results were analyzed using HALO^TM^ software (version 3.6.4134.464). First, we trained the machine for region identification, focusing on tumor parenchyma and stroma (Figure 2C). We then set the threshold for nuclear identification and recognized nuclei (Figure 2D), followed by the identification of positive cells in each channel based on defined thresholds (Figure 2E). Finally, as shown in Figure 2F,G, we obtained identification and quantification of different phenotypic cell subpopulations in distinct regions.

**Figure 3:**
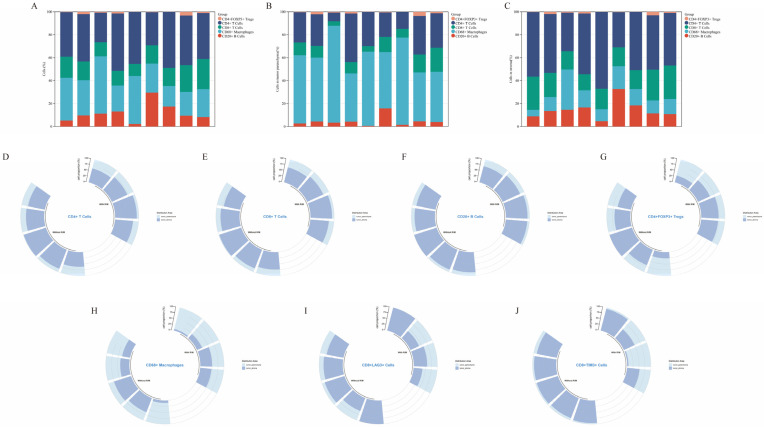
Percentage of each infiltrating immune cell type within entire field of view (**A**), within the tumor parenchyma (**B**), and within the tumor stroma (**C**). Panels (**D**–**J**) show the distribution of various immune cell types in both the tumor parenchyma and stroma. With R/M, with recurrent/metastatic patients; Without R/M, without recurrent/metastatic patients.

Figure 3A–C show the percentage of various types of infiltrating immune cells across the nine patient samples. Across the whole tissue section, the infiltrating immune cells were predominantly CD4+ T cells and CD68+ macrophages (Figure 3A). In the tumor parenchyma, the infiltrating immune cells were primarily CD68+ macrophages (Figure 3B); while in the stroma, CD4+ T cells were dominant (Figure 3C). In terms of infiltrating immune cell distribution, CD20+ B cells, CD8+ T cells, CD4+ T cells, and CD4+ FoxP3+ Treg cells were mainly distributed in the stroma (Figure 3D–G), whereas CD68+ macrophages were concentrated in the tumor parenchyma (Figure 3H). This distribution pattern was consistent across both recurrent/metastatic and non-recurrent/non-metastatic patients. However, CD8+LAG3+ (Figure 3I) and CD8+TIM3+ cells (Figure 3J) were mainly located in the tumor parenchyma of recurrent/metastatic patients, while in non-recurrent/non-metastatic patients, these cells were predominantly found in the stroma.

**Figure 4:**
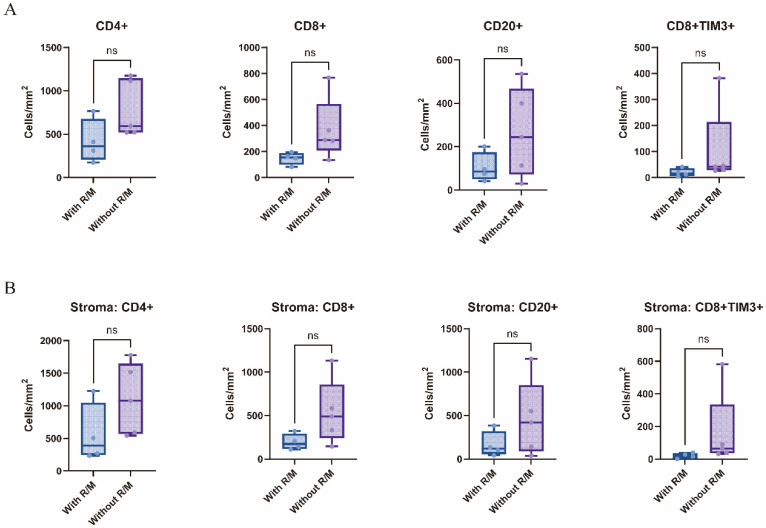
Differences in infiltrating immune cells between recurrent/metastatic and non-recurrent/non-metastatic patients in the whole-section view (**A**) and the tumor stroma (**B**). With R/M, with recurrent/metastatic patients; Without R/M, without recurrent/metastatic patients. ns.—*p* > 0.05.

An analysis of immune cell infiltration density across the whole slide, as well as within the tumor parenchyma and stroma, revealed certain trends. Compared to the non-recurrent/non-metastatic group, the recurrent/metastatic group exhibited lower densities of CD4+ T cells, CD8+ T cells, and CD20+ B cells, particularly within the stromal region (*p* > 0.05, Figure 4). In contrast, no significant differences were observed in the infiltration densities of CD68+ macrophages and CD4+ FoxP3+ Treg cells between the two groups. Regarding immune checkpoint density, CD8+TIM3+ cells were also less abundant in the recurrent/metastatic group compared to the non-recurrent/non-metastatic group, with a notable reduction observed in the tumor stroma (*p* > 0.05, Figure 4).

### 3.3. Tertiary Lymphoid Structures (TLSs)

**Figure 5:**
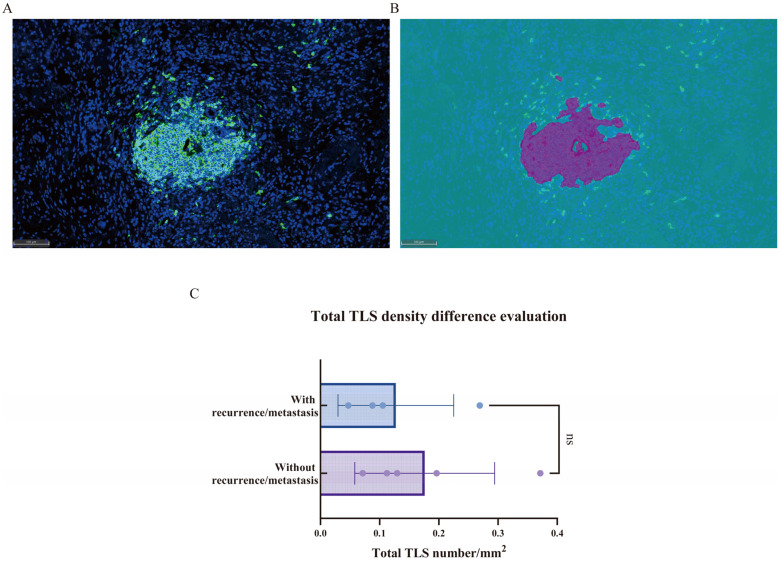
(**A**,**B**) Schematic representation of tertiary lymphoid structures. The multiplex immunofluorescence staining image (**A**); the corresponding HALO^TM^ software model (**B**). (**C**) Comparison of tertiary lymphoid structures between recurrent/metastatic and non-recurrent/non-metastatic patients. ns.—*p* > 0.05.

As shown in Figure 5A,B, TLSs were defined as clusters of more than 200 CD20+ B cells. Compared to recurrent/metastatic patients, non-recurrent/non-metastatic patients exhibited a higher density of tertiary lymphoid structures across the whole slide, although this difference was not statistically significant (*p* > 0.05, Figure 5C).

### 3.4. Nearest Neighbor Analysis

**Figure 6:**
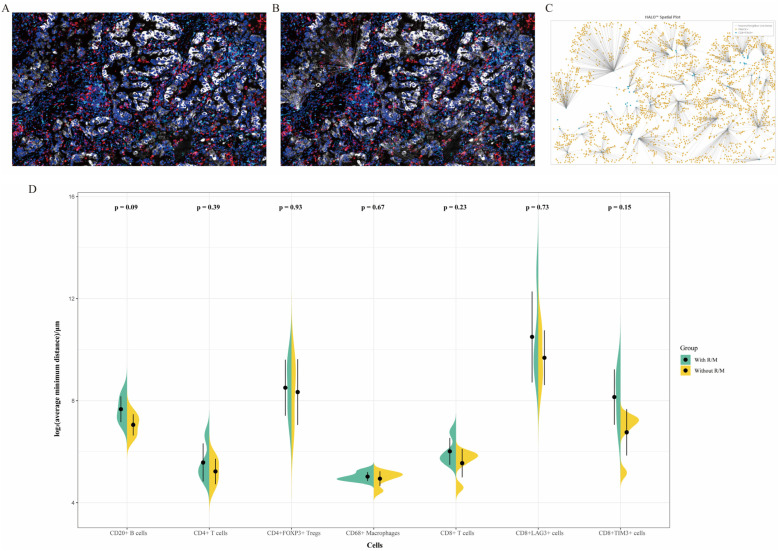
(**A**–**C**) Schematic representation of the nearest neighbor analysis for CD8+TIM3+ cells in Panel 2. The multiplex immunofluorescence staining image (**A**); the in situ map from HALO^TM^ analysis (**B**); and the HALO^TM^ model (**C**). (**D**) Comparison of the average minimum distances between various infiltrating immune cells and tumor cells in recurrent/metastatic versus non-recurrent/non-metastatic patients. With R/M, with recurrent/metastatic patients; Without R/M, without recurrent/metastatic patients.

Additionally, we performed a nearest neighbor analysis (Figure 6A–C) to identify the immune cells and immune checkpoints closest to Pan-CK+ tumor cells across the entire slide, calculating the average distance. As shown in Figure 6D, CD20+ B cells, CD8+ cells, and CD8+TIM3+ cells tended to be closer to tumor cells in the non-recurrent/non-metastatic group compared to the recurrent/metastatic group, although this difference did not reach statistical significance (*p* > 0.05).

### 3.5. Factors Associated with Survival

The univariate Cox regression analysis of disease-free survival (DFS) based on clinicopathological characteristics and the immune microenvironment identified several factors with potential prognostic significance, though none reached statistical significance (0.05 < *p* < 0.1). As shown in Table 2, the density of CD8+TIM3+ cells within the whole slide (HR = 0.89) or stromal region (HR = 0.91) suggested a potential association with improved DFS. Additionally, the density of TIM3+ cells throughout the whole slide and in the stroma, as well as the density of CD8+LAG3- cells in the stroma, demonstrated a slight reduction in hazard (HR = 0.99). In contrast, the average minimum distance of CD8+TIM3+ cells to tumor cells was associated with a slight increase in hazard (HR = 1.01).

These findings suggest that certain immune microenvironment factors, particularly those related to the expression of immune checkpoints TIM3 and LAG3, may influence DFS. However, further investigation is needed to confirm these associations.

## 4. Discussion

Our study offers a comprehensive analysis of the immune microenvironment in NSCLC patients harboring *MET* exon 14 skipping mutations, revealing the distinct immune profiles associated with recurrence and metastasis. By utilizing mIF and analyzing key immune markers such as CD4, CD8, CD20, and PD-L1, we have elucidated significant differences in immune cell distribution and immune checkpoint expression between recurrent/metastatic and non-recurrent/non-metastatic cases.

One of the primary findings of this study is the reduced density of infiltrating immune cells, including CD4+ T cells, CD8+ T cells, and CD20+ B cells, in the recurrent/metastatic group, particularly within the tumor stroma. This suggests that a lower presence of these immune cells may contribute to tumor progression and a poorer prognosis in these patients. Previous studies have established the significance of tumor-infiltrating lymphocytes (TILs) in cancer progression. In various types of cancer, TILs can serve as predictors of treatment response and prognosis. For instance, in triple-negative breast cancer (TNBC), patients with low expression of the CD247 and CD4 gene phenotypes tend to have poorer outcomes [17]. Similarly, high levels of CD8+ TILs in NSCLC are associated with better prognosis [18]. Furthermore, stromal tumor-infiltrating lymphocytes (sTILs) have been linked to PFS and OS in metastatic breast cancer (MBC) patients undergoing chemotherapy [19]. A recent single-cell transcriptomic study of NSCLC patients harboring rare driver mutations treated with anti-PD-1 therapy identified GZMK+ CD8 effector memory T cells as critical mediators of effective antitumor responses, whereas myeloid cells were implicated in promoting immunosuppressive mechanisms [20]. In addition, TILs have been recognized as effective biomarkers for predicting the efficacy of ICIs in NSCLC patients [21,22]. Our findings further support this, emphasizing the potential of immune cells in regulating tumor behavior and patient prognosis.

Furthermore, no significant difference was observed in the overall density of immune checkpoint markers, including TIM3 and LAG3, between the two groups. However, a significant reduction in CD8+TIM3+ cells within the stroma was detected in the recurrence/metastasis group, potentially reflecting differences in CD8+ cell density between the groups. Notably, CD8+TIM3+ and CD8+LAG3+ cells were predominantly located in the tumor parenchyma of patients with recurrence/metastasis, whereas these cells were mainly distributed in the stromal regions of non-recurrent/non-metastatic patients. CD8+TIM3+ T cells are generally associated with T cell exhaustion, characterized by functional inhibition, limited proliferative capacity, and reduced cytotoxicity [23]. A study by Yang G et al. demonstrated that a high density of dysfunctional CD8+ T cells (CD8+TIM3+ T_dys_) in the invasive margin (IM) was significantly correlated with lymph node metastasis and poorer recurrence-free survival (RFS) in NSCLC [24]. Similarly, other studies have shown that LAG3 contributes to T cell exhaustion by synergizing with other co-inhibitory receptors, thereby impairing their antitumor activity [25]. In PD-L1-positive NSCLC, elevated levels of LAG3+CD8+ T cells may be associated with reduced efficacy of ICIs [26]. In our cohort, the most frequently co-mutated gene was TP53, occurring in 25% of patients, which is consistent with previously reported frequencies of approximately 30% [27]. Interestingly, a study evaluating first-line immunotherapy in NSCLC patients with MET exon 14 skipping mutations found that TP53-mutated tumors showed evidence of a numerically enhanced ORR and prolonged PFS, along with an advantage in OS when analyzed in a multivariable context, compared to non-mutated cases [16]. These observations suggest that TP53 mutation status may carry prognostic or predictive significance in the context of immunotherapy for METex14-altered NSCLC.

Another intriguing aspect of our study is the analysis of TLSs. We observed a higher density of TLSs in non-recurrent/metastatic patients harboring the *MET* exon 14 skipping mutation, although this difference was not statistically significant. Several studies have demonstrated that the presence of TLSs in NSCLC patients is associated with better clinical outcomes [28,29,30,31,32]. Furthermore, the formation of TLSs not only impacts prognosis but also plays a pivotal role in immunotherapy. Research suggests that higher TLS density is correlated with improved responses to ICIs [29,33,34], such as PD-1 blockers, in NSCLC patients. Notably, a recent study proposed novel molecular subtypes of MET exon 14 skipping NSCLC, among which the immune-activated subtype was associated with the most favorable long-term survival. This subtype was characterized by increased formation of TLSs, spatial cooption of PD-L1+ cancer cells, and enrichment of GZMK+ CD8+ T cells—features indicative of a more robust antitumor immune response. These findings suggest that TLSs may serve as a promising prognostic and predictive biomarker in MET exon 14 skipping NSCLC [35]. The absence of a statistically significant difference in our cohort may be due to sample size limitations, and further studies are needed to clarify the role of TLSs in *MET* exon 14 skipping mutation-positive NSCLC.

Additionally, the nearest neighbor analysis revealed that immune cells, particularly CD20+ B cells and CD8+ T cells, were in closer proximity to tumor cells in the non-recurrent/non-metastatic group compared to the recurrent/metastatic group. This spatial relationship may suggest a more active immune surveillance mechanism in non-recurrent patients, preventing tumor progression. Multiple studies have demonstrated that the proximity of CD8+ T cells to tumor cells and their spatial distribution within the tumor microenvironment hold significant prognostic value across various cancer types [24,36,37,38]. A study on spatial patterns of cells with prognostic impact in the tumor microenvironment of NSCLC found that the proximity between CD20+ B cells and N1-like neutrophils was significantly associated with longer DFS.

While our findings highlight potential prognostic markers within the immune microenvironment of *MET* exon 14 skipping mutation-positive NSCLC, certain limitations should be acknowledged. First, the small sample size, particularly in the mIF analysis, may limit the statistical power of our findings. Second, the retrospective nature of this study restricts our ability to establish causal relationships between immune cell infiltration and patient outcomes. Third, due to technical constraints of mIF, the number of markers that can be simultaneously assessed on a single FFPE section is limited; thus, other important immune checkpoints such as CTLA-4 and TIGIT could not be included in the current analysis. In addition, the use of archived FFPE samples made it infeasible to perform flow cytometry or PCR-based assays to evaluate immune-related gene expression, including CXCR3, IFN-γ, and TNF-α. These limitations restricted our ability to conduct more comprehensive immunophenotyping and functional analyses. Future prospective studies with larger cohorts, fresh tissue specimens, and expanded panels incorporating additional immune checkpoints and cytokine profiling are warranted to validate our findings and to further elucidate the prognostic and therapeutic relevance of the immune microenvironment in this patient population.

## 5. Conclusions

In this study, we investigated the immune microenvironment of NSCLC patients harboring *MET* exon 14 skipping mutations using mIF staining. Our findings reveal distinct patterns of immune cell distribution between recurrent/metastatic and non-recurrent/non-metastatic patients and suggest that immune checkpoints may play a critical role in influencing DFS in this subset of NSCLC patients, warranting further research to confirm these associations and explore novel therapeutic strategies.

## Figures and Tables

**Table 1:** Clinicopathological differences between recurrent/metastatic and non-recurrent/non-metastatic groups.

Characteristic	With Recurrence/Metastasis	Without Recurrence/ Metastasis	*p* Value
	*n* = 6 (%)	*n* = 51 (%)	
Age (years)			0.665
≤60	1 (16.7)	15 (29.4)	
>60	5 (83.3)	36 (70.6)	
Gender			0.025
Male	6 (100.0)	24 (47.1)	
Female	0 (0.0)	27 (52.9)	
Smoking status			0.000
Current smokers	0 (0.0)	13 (25.5)	
Former smokers	5 (83.3)	2 (3.9)	
Never smokers	1 (16.7)	36 (70.6)	
T stage			0.000
T1a	0 (0.0)	8 (15.7)	
T1b	0 (0.0)	25 (49.0)	
T1c	1 (16.7)	13 (25.5)	
T2a	3 (50.0)	1 (2.0)	
T2b	1(16.7)	1 (2.0)	
T3	0 (0.0)	2 (3.9)	
T4	1(16.7)	1 (2.0)	
N stage			0.194
N0	4 (66.7)	45 (86.0)	
N1	2 (33.3)	4 (10.5)	
N2	0 (0.0)	2 (3.5)	
Histology			0.731
Adenocarcinoma in situ	0 (0.0)	1 (2.0)	
Minimally invasive adenocarcinoma	0 (0.0)	8 (15.7)	
Invasive adenocarcinoma	6 (100.0)	39 (76.5)	
Squamous cell carcinoma	0 (0.0)	1 (2.0)	
Adenocarcinoma with sarcomatoid carcinoma	0 (0.0)	2 (3.9)	
Intravascular carcinoma embolus			0.044
Present	3 (50.0)	6 (11.8)	
Absent	3 (50.0)	45 (88.2)	
Pleural infiltration			0.060
Present	3 (50.0)	7 (13.7)	
Absent	3 (50.0)	44 (86.3)	
PD-L1 tumor proportion scale			0.631
≥50%	3 (50.0)	11 (21.6)	
≥1% and <50%	1 (16.7)	8 (15.7)	
<1%	2 (33.3)	21 (41.2)	
Unknown	0 (0.0)	11 (21.6)	

**Table 2:** Univariate Cox regression analysis of disease-free survival (DFS) based on clinicopathological characteristics and the immune microenvironment.

Variables	HR (95%CI)	*p*-Value
Age	0.78 (0.59–1.05)	0.099
Density of TIM3+ cells	0.99 (0.99–1.00)	0.089
Density of CD8+TIM3+ cells	0.89 (0.80–1.01)	0.064
Density of TIM3+ cells in the stroma	0.99 (0.99–1.00)	0.058
Density of CD8+TIM3+ cells in the stroma	0.91 (0.83–1.01)	0.078
Density of CD8+LAG3- cells in the stroma	0.99 (0.98–1.00)	0.095
Average minimum distance of CD8+TIM3+ cells	1.01 (1.00–1.02)	0.064

HR: Hazard Ratio, CI: Confidence Interval.

## Data Availability

The original contributions presented in this study are included in the article/Supplementary Material. Further inquiries can be directed to the corresponding author.

## Supplementary Materials

The following supporting information can be downloaded at https://www.mdpi.com/article/10.3390/curroncol32070403/s1, Table S1: Final, optimized six-plex mIF assay conditions for panel 1; Table S2: Final, optimized six-plex mIF assay conditions for panel 2; Table S3: Clinicopathologic characteristics of the patients with MET exon 14 skipping mutations.
