# Supplementary material for: MET Exon 14 Skipping Mutations in Lung Cancer: Clinical–Pathological Characteristics and Immune Microenvironment
Source: Curr Oncol. 2025 Jul 14;32(7):403. doi: 10.3390/curroncol32070403 (PMC12293242; doi:10.3390/curroncol32070403)
Supplement: Supplementary file 1 [file curroncol-32-00403-s001.zip › curroncol-3693924-supplementary.pdf]

Supplementary Table S1. Final, optimized six-plex mIF assay conditions for panel 1.

Staining order	Primary antibody	Provider	Identifier	Dilution	Incubation (min)	Secondary antibody	TSA-Opal
1	CD4	Abcam	ab133616	1:500	60	ARH1001EA(Akoya)	520
2	CD20	Zsbio	ZM-0039	NA	30	ARH1001EA(Akoya)	570
3	CD68	Abcam	ab213363	1:500	45	ARH1001EA(Akoya)	690
4	CD8	Abcam	ab101500	1:200	60	ARH1001EA(Akoya)	480
5	FOXP3	Abcam	ab191416	1:100	60	ARH1001EA(Akoya)	620
6	Panck	Abcam	ab7753	1:1000	20	ARH1001EA(Akoya)	DIG 780

Supplementary Table S2. Final, optimized six-plex mIF assay conditions for panel 2.

Staining order	Primary antibody	Provider	Identifier	Dilution	Incubation (min)	Secondary antibody	TSA-Opal
1	CD20	Zsbio	ZM-0039	NA	30	ARH1001EA(Akoya)	520
2	CD8	Abcam	ab101500	1:200	60	ARH1001EA(Akoya)	480
3	PD-L1	CST	Cst13684S	1:2000	45	ARH1001EA(Akoya)	570
4	TIM3	Abcam	ab241332	1:1000	60	ARH1001EA(Akoya)	690
5	LAG3	Abcam	ab180187	1:100	60	ARH1001EA(Akoya)	620
6	Panck	Abcam	ab7753	1:1000	20	ARH1001EA(Akoya)	DIG 780

Supplementary Table S3. Clinicopathologic characteristics of the patients with *MET* exon 14 skipping mutations.

Characteristic	*n = 57 (%)*
**Age (years)**	
≤60	16 (28.1)
＞60	41 (71.9)
**Gender**	
Male	30 (52.6)
Female	27 (47.4)
**Smoking status**	
Current smokers	13 (22.8)
Former smokers	7 (12.3)
Never smokers	37 (64.9)
**T stage**	
T1a	8 (14.0)
T1b	25 (43.9)
T1c	14 (24.6)
T2a	4 (7.0)
T2b	2 (3.5)
T3	2 (3.5)
T4	2 (3.5)
**N stage**	
N0	49 (86.0)
N1	6 (10.5)
N2	2 (3.5)
**Metastatic disease during follow-up**	
Yes	6 (10.5)
No	51 (89.5)
**Histology**	
Adenocarcinoma in situ	1 (1.8)
Minimally invasive adenocarcinoma	8 (14.0)
Invasive adenocarcinoma	45 (78.9)

Squamous cell carcinoma	1 (1.8)
Adenocarcinoma with sarcomatoid carcinoma	2 (3.5)
**Intravascular carcinoma embolus**	
Present	9 (15.8)
Absent	48 (84.2)
**Pleural infiltration**	
Present	10 (17.5)
Absent	47 (82.5)
**PD-L1 tumor proportion scale**	
≥50%	14 (24.6)
≥1%＜50%	9 (15.8)
＜1%	23 (40.3)
Unknown	11 (19.3)
